# Self-reported Access to Firearms Among Patients Receiving Care for Mental Health and Substance Use

**DOI:** 10.1001/jamahealthforum.2021.1973

**Published:** 2021-08-06

**Authors:** Julie E. Richards, Elena Kuo, Christine Stewart, Jennifer F. Bobb, Kayne D. Mettert, Ali Rowhani-Rahbar, Marian E. Betz, Rebecca Parrish, Ursula Whiteside, Jennifer M. Boggs, Gregory E. Simon

**Affiliations:** 1 Health Research Institute, Kaiser Permanente Washington, Seattle; 2 Department of Health Services, University of Washington, Seattle; 3 Department of Epidemiology, University of Washington School of Public Health, Seattle; 4 Harborview Injury Prevention and Research Center, Seattle, Washington; 5 Department of Emergency Medicine, University of Colorado School of Medicine, Aurora; 6 Department of Mental Health & Wellness, Kaiser Permanente Washington, Seattle; 7 NowMattersNow.org, Seattle, Washington; 8 Psychiatry and Behavioral Sciences, University of Washington, Seattle; 9 Institute for Health Research, Kaiser Permanente Colorado, Aurora

## Abstract

**Importance:**

Firearms are the most common method of suicide, one of the “diseases of despair” driving increased mortality in the US over the past decade. However, routine standardized questions about firearm access are uncommon, particularly among adult populations, who are more often asked at the discretion of health care clinicians. Because standard questions are rare, patterns of patient-reported access are unknown.

**Objective:**

To evaluate whether and how patients self-report firearm access information on a routine mental health monitoring questionnaire and additionally to examine sociodemographic and clinical associations of reported access.

**Design, Setting, and Participants:**

Cross-sectional study of patients receiving care for mental health and/or substance use in primary care or outpatient mental health specialty clinics of Kaiser Permanente Washington, an integrated health insurance provider and care delivery system.

**Main Outcomes and Measures:**

Electronic health records were used to identify patients who completed a standardized self-reported mental health monitoring questionnaire after a single question about firearm access was added from January 1, 2016, through December 31, 2019. Primary analyses evaluated response (answered vs not answered) and reported access (yes vs no) among those who answered, separately for patients seen in primary care and mental health. These analyses also evaluated associations between patient characteristics and reported firearm access. Data analysis took place from February 2020 through May 2021.

**Results:**

Among patients (n = 128 802) who completed a mental health monitoring questionnaire during the study period, 74.4% (n = 95 875) saw a primary care clinician and 39.3% (n = 50 631) saw a mental health specialty clinician. The primary care and mental health samples were predominantly female (63.1% and 64.9%, respectively) and White (75.7% and 77.0%), with a mean age of 42.8 and 51.1 years. In primary care, 83.4% of patients answered the question about firearm access, and 20.9% of patients who responded to the firearm question reported having access. In mental health, 91.8% of patients answered the question, and 15.3% reported having access.

**Conclusions and Relevance:**

In this cross-sectional study of adult patients receiving care for mental health and substance use, most patients answered a question about firearm access on a standardized mental health questionnaire. These findings provide a critical foundation to help advance understanding of the utility of standardized firearm access assessment and to inform development of practice guidelines and recommendations. Responses to standard firearm access questions used in combination with dialogue and decision-making resources about firearm access and storage may improve suicide prevention practices and outcomes.

## Key Points

**Question:**

Did patients respond to a standard question about firearm access on a mental health questionnaire, and, if so, how did they respond?

**Findings:**

In this cross-sectional study of 128 802 patients receiving care for mental health and substance use, 83% of primary care patients answered a standard question about firearm access and 21% reported access. In mental health clinics, 92% of patients answered the question and 15% reported access.

**Meaning:**

In this study, most patients reported firearm access on standard questionnaires; this screening practice may improve efforts to identify and engage patients at risk of suicide in discussions about securing firearms.

## Teaser

This cross-sectional study evaluates whether and how patients self-report firearm access information on a routine mental health monitoring questionnaire and examines sociodemographic and clinical associations of reported access.

## Introduction

Firearms are the most common method of suicide, one of the “diseases of despair” driving increased mortality and decreased life expectancy in the US over the past decade.^1,2,3^ Firearms are highly lethal, with case fatality rates of 85% to 95%.^2^ Likewise, suicide accounts for the majority of firearm deaths in the US (60% nationally; state-level range, 38%-92%),^2^ particularly among adult populations. However, few health care organizations routinely assess firearm access using standardized questions; rather, they rely on clinician discretion to query patients.^4^ Relying on clinician discretion to ask patients about firearms access undoubtedly results in missing or incomplete information,^5,6^ especially considering that clinicians often have limited time to solicit information and develop a care plan with patients.^7,8^ Firearm access is more commonly assessed in pediatric settings, but follow-up practices are variable.^4,9^ Patients presenting to emergency settings with suicidality typically have the highest rates of firearm access assessment. Prior studies have reported 50% or higher assessment rates: 76% of individuals involuntarily hospitalized as a “danger to self” (per California law),^10^ 74.5% of pediatric emergency department patients with suicidal ideation,^11^ 69.9% of patients accessing an urban psychiatric emergency service,^12^ 57% of patients presenting with a suicide attempt in Winnipeg emergency departments,^13^ and 50% of all patients reporting suicidal ideation in 8 US emergency departments.^6^ Patients presenting to primary care (PC) and/or outpatient mental health (MH) specialty settings with suicidality are much less likely to be assessed for firearm access. One study found that only one-third of patients reporting suicidal thoughts in the prior 2 weeks were assessed for firearm access,^14^ and another reported similar rates, even among patients with recent emergency care visits for suicidality.^15^

Implementing standardized firearm access questions can likely increase firearm access identification; for example, a retrospective medical record review of hospitalized psychiatric patients found that reporting access to firearms increased from 1% to 9% after implementing routine firearm screening.^16^ Because using standard questions to routinely assess firearm access among adult patients is rare, we have limited knowledge as to whether and how patients might answer standard population-based firearm access questions. To our knowledge, no studies have reported nonresponse rates, examined associations of routine patient-reported firearm access, or explored longitudinal consistency of self-reported access. Advancing our understanding of how patients answer such questions is critical for assessing the utility of this practice for supporting suicide prevention practices in health care settings, such as collaborative discussions about lethal means safety.^17,18^

The primary goal of this study was to understand whether and how patients receiving care for MH and substance use responded to a single question about firearm access after it was added to a standardized self-reported MH monitoring questionnaire by a large regional health care system. Specifically, we described the proportion of patients who answered the firearm access question and the proportion who reported access among those who answered, in both PC and outpatient MH specialty settings. Additionally, we described associations between patient-reported access to firearms in these settings and sociodemographic and clinical characteristics known to be associated with suicide attempt. Secondarily, we explored consistency in patient-reported access to firearms among patients with 2 or more visits over the 4-year observation period.

## Methods

The Kaiser Permanente Institutional Review Board approved this study and waived the need for patient informed consent and Health Insurance Portability and Accountability Act authorization for access, use, and collection of protected health information from medical records to conduct this study, because use of the protected health information involved no more than a minimal risk to the privacy of individuals. This study followed the Strengthening the Reporting of Observational Studies in Epidemiology (STROBE) reporting guideline.

### Data Sources and Patient Sample

Data were collected from Kaiser Permanente Washington, a large integrated health insurance provider and care delivery system in Washington State, serving approximately 700 000 mostly urban/suburban enrollees, that routinely collects and records patient-reported firearm access in electronic health records (EHRs). Data regarding responses to MH questionnaires were extracted from EHR databases, and data regarding MH diagnoses, co-occurring medical diagnoses, and treatment history were extracted from both EHR databases and insurance claims.

**Figure 1.**
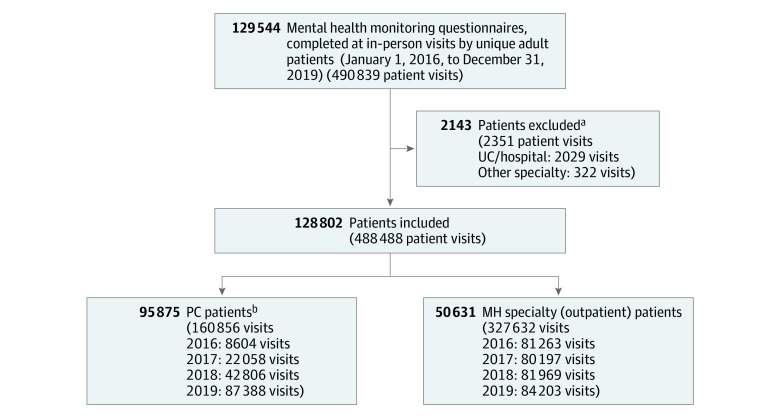
Analytic Sample Adult outpatient visits with documentation of a mental health monitoring questionnaire during the study period of January 1, 2016, through December 31, 2019. ^a^Patient visits outside primary care (PC) and outpatient mental health (MH) were excluded, including urgent care (UC) visits and hospitalizations, and other specialty care visits, because this questionnaire was rarely completed in those settings (<1% of all questionnaires). ^b^Includes 17 704 patients with at least 1 visit to both the PC and MH specialty settings during the study period.

Beginning in August 2015, a question about firearm access was added to an MH monitoring questionnaire (eFigure 1 in the Supplement) as part of all in-person visits to outpatient MH clinics. The questionnaire was later integrated into PC as part of an MH integration program. Specifically, 22 PC clinics implemented new workflows between 2016 and 2018 (7 waves 4 months apart)^19^ to support improved care for depression, suicidality,^20^ and substance use.^21,22^ New workflows included an EHR-based previsit reminder (eFigure 2 in the Supplement), which prompted PC clinic staff to ask adult patients (≥18 years) with a current depression or substance use disorder diagnosis to complete the monitoring questionnaire on paper during the appointment rooming process. Patient responses were typically documented in the EHR immediately prior to the visit to guide clinical care. The final analytic sample included all questionnaires completed at in-person visits to a PC or MH clinician over a 4-year period, January 1, 2016, through December 13, 2019 (eAppendix in the Supplement), during which time the questionnaire was routinely administered in the MH specialty but was more slowly integrated into PC workflows (Figure 1). Kaiser Permanente Washington performance metrics (developed to support suicide prevention)^23^ indicated that in 2019 (following MH integration), 97% of MH specialty visits and 27% of PC visits had a documented MH monitoring questionnaire.

### Firearm Access

Patient-reported firearm access was evaluated in 2 stages: (1) response (ie, answered vs not answered) to a standard question, “Do you have access to guns?” documented during a PC or MH specialty visit (hereafter “eligible visits”) and (2) reported access (answered “Yes” vs answered “No”). Nonresponse to the firearm question was defined by identifying documented completed questionnaires using answered questions unique to this MH monitoring questionnaire that lacked a response to the firearm question.

### Sociodemographic Characteristics

Sociodemographic characteristics known to be associated with firearm ownership and suicide risk^24,25^ were extracted using health system data at the time of each patient visit, including age (continuous), sex (male/female), race and ethnicity self-reported by study participants (American Indian/Alaska Native, Asian, Black, Hawaiian/Pacific Islander, Hispanic/Latinx, White, other, unknown), and insurance type (commercial, Medicare, Medicaid, not enrolled). Rurality was defined using national county-level data^26^ and 4 categories (urban, large suburban, small suburban, mostly rural) previously used to examine firearm mortality trends.^27^

### MH and Substance Use Characteristics

Mental health symptom severity and substance use frequency, collected on the same questionnaire as firearm access, were used to describe the PC and MH patient populations and examine associations with response to the firearm access question. Severity of depressive symptoms was defined using the total score from the 9-item Patient Health Questionnaire (PHQ-9) (0-4, minimal or none; 5-9, mild; 10-14, moderate; 15-19, moderately severe; 20-24, severe),^28,29^ and frequency of suicidal ideation derived from response options for the PHQ-9 ninth question assessing frequency of thoughts about self-harm (0, not at all; 1, several days; 2, more than half the days; 3, nearly every day).^30,31^ Presence of anxiety symptoms was defined using the Generalized Anxiety Disorder 2-item (GAD-2; score, 3-6).^32^ Alcohol consumption was defined based on gender-specific cut points of the Alcohol Use Disorders Identification Test–Consumption (AUDIT-C)^33,34^ (nondrinking: score, 0; low level: score, 1-2 for women, 1-3 for men; moderate level: score, 3-7 for women, 4-7 for men; high level: score, 8-12).^35^ Cannabis use and other illicit drug use were derived from the response options for 2 single-item questions assessing frequency of use.^21^ Patient populations were also described using *International Statistical Classification of Diseases, Tenth Revision, Clinical Modification* diagnostic codes for depression, anxiety, serious mental illness (bipolar, schizophrenia, other psychosis, or personality disorders), substance use disorders, suicide attempt, and history of medical comorbidity associated with mortality using the Charlson comorbidity index score^36,37^ in the 365 days prior to firearm access assessment.

### Statistical Analysis

Descriptive statistical analyses summarized patient characteristics using data from the first eligible in-person visit to a PC and/or MH clinic during the study period. Primary analyses evaluated associations between sociodemographic and clinical characteristics and reported firearm access in 2 stages: stage 1 descriptively evaluated firearm question response (answered/not answered); stage 2 descriptively evaluated firearm access reported among those who answered (yes/no). Analyses were stratified by care setting owing to variation in MH monitoring questionnaire administration workflows and potential differences in response by care setting. Logistic regression models estimated the odds ratio (OR) of response (answered/not answered) and reported access across sociodemographic, MH, and substance use characteristics associated with suicide attempt.^24^ Unadjusted (univariate) analyses and mutually adjusted (multivariate) regression analyses were conducted. In multivariate models, the following variables were included: age, sex, race and ethnicity, rurality, and prior-year diagnoses (additionally adjusted for prior-year enrollment status). Adjusted analyses did not include insurance owing to strong correlation with age and sex, nor patient-reported symptoms nor substance use owing to strong correlation with prior-year MH diagnoses and because inclusion would require further limiting the sample to individuals who answered all these questions (a sample more likely to also answer the firearm question). Sensitivity analyses repeated the primary analyses to examine whether using all visits in the study period (multiple visits for individual patients) potentially changed the results, using generalized estimating equations with an independence working correlation structure.^38^ In all models, CIs were set as 95% (α = .05) using a 2-tailed distribution, and SEs were calculated using the robust sandwich estimator.

Secondarily, we explored firearm question response and access consistency graphically using all eligible visits among patients in the sample who completed the MH monitoring questionnaire more than once. We repeated the staged approach to separately describe consistency in response (answered/not answered) and patient-reported firearm access (access/no access). Stage 1 used the response from the first visit in the study period and a composite measure of all responses at subsequent visits to describe the proportions of patients who always, sometimes, or never responded to the question about firearm access. Stage 2 was further limited to patients who answered the firearm access question at least twice, and similarly used data from the first visit and a composite measure of all subsequent visits to describe the proportions of patients who always, sometimes, or never reported access to firearms. These analyses further explored response consistency for patients with differing lengths of time between their first and last visits and differing numbers of visits in the study period. All analyses were performed using Stata/MP, version 15.1 (StataCorp LLC).^39^

## Results

### Patient Characteristics

Among all patients (n = 128 802) who completed an MH monitoring questionnaire during the study period, 95 875 (74.4%) saw a PC clinician and 50 631 (39.3%) saw an MH specialty clinician (17 704 patients [13.7%] saw both). The PC and MH samples were predominantly female (64.9% and 63.1%, respectively), White (77.0% and 75.7%), and lived in an urban area (33.3% and 42.1%). Among the MH sample, 52.4% reported moderate to severe depressive symptoms (PHQ-9 score ≥10), and 25.4% reported some suicidal ideation (PHQ-9 ninth question score 1-3). Among the PC population, 27.9% reported moderate to severe depressive symptoms, and 11.1% reported some suicidal ideation (Table 1).

**Table 1.** Patient Characteristics^a^

Characteristic	Patients, %	
	PC (n = 95 875)	MH (n = 50 631)
Age, mean (SD), y	51.1 (18.4)	42.8 (17.4)
Age category		
18-39 y	30.9	49.7
40-64 y	42.3	36.6
≥65 y	26.8	13.7
Sex^b^		
Female	64.9	63.1
Male	35.1	36.9
Race and ethnicity		
American Indian/Alaska Native	1.7	2.1
Asian	6.1	5.4
Black	4.1	4.6
Hawaiian/Pacific Islander	0.9	1.1
Hispanic/Latinx	4.7	5.5
Other/unknown^c^	5.4	5.6
White	77.0	75.7
Insurance^b^		
Commercial	55.6	72.6
Medicare	23.2	17.7
Medicaid	5.0	6.2
Not enrolled	16.5	3.5
Rural/urban^d^		
Urban	33.3	42.1
Suburban		
Large	22.1	26.0
Smaller	41.9	28.4
Mostly rural	2.8	3.5
Alcohol consumption (AUDIT-C)^e^		
Never	33.0	31.3
Low level	40.7	40.0
Moderate level	24.4	26.1
High level	1.8	2.5
Cannabis use^e^		
None	76.1	65.0
Weekly-monthly	17.9	24.3
Daily/almost daily	5.9	.6
Other drug use^e^		
None	97.5	94.3
Daily-monthly	2.5	5.7
Depressive symptoms (PHQ-9)^e^		
None-minimal	48.5	21.5
Mild	23.6	26.0
Moderate	14.4	22.3
Moderate-severe	8.6	17.0
Severe	4.9	13.1
Suicidal ideation (PHQ-9 question 9)^e^		
Not at all	88.9	74.6
Several days	7.8	15.8
More than half	2.1	5.5
Nearly every day	1.2	4.1
Anxiety symptoms (GAD-2)^e^		
Negative	69.9	42.3
Positive	30.1	57.7
Depression diagnosis^f^		
No	60.9	36.7
Yes	39.1	63.3
Anxiety diagnosis^f^		
No	61.1	22.8
Yes	38.9	77.2
Serious mental illness diagnosis^f^		
No	97.4	86.4
Yes	2.6	13.6
Substance use disorder diagnosis^f^		
No	92.6	85.5
Yes	7.4	14.5
Suicide attempt diagnosis^f^		
No	99.8	98.9
Yes	0.2	1.1
Charlson Comorbidity Index score^g^		
0-1	84.0	88.3
≥2	16.0	11.7

Abbreviations: AUDIT-C, Alcohol Use Disorders Identification Test–Consumption; GAD-2, Generalized Anxiety Disorder 2-item; MH, mental health; PC, primary care; PHQ-9, Patient Health Questionnaire.

^a^ Time-varying information defined at the time of the first outpatient MH and PC visit during the study period. Includes information about 17 704 patients seen in both settings. Means presented for continuous age variables and proportions presented for categorical variables for patients with data available.

^b^ The “not enrolled” insurance category includes patients of a Spokane-based health care system acquired by Kaiser Permanente Washington during the study period. Sex missing for 1 MH patient.

^c^ “Other” race and ethnicity includes responses that do not fall into 1 of the broader categories, such as “Irish,” “Ashkenazi Jewish,” or “human race.”

^d^ Based on a condensed version of the 2013 National Center for Health Statistics (NCHS) county urban-rural categorization. Urban (NCHS: large central metro), large suburban (NCHS: large fringe metro; described as a large suburban area in data briefs), smaller suburban (NCHS: medium metro and small metro), and most rural (NCHS: micropolitan and noncore). Missing for 273 MH patients and 275 PC patients.

^e^ Recorded via self-report on the MH monitoring questionnaire. See eTable 1 in the Supplement for a summary of nonresponse.

^f^ Diagnosis in year prior (including this visit with an MH questionnaire); serious mental illness diagnoses include bipolar, schizophrenia, other psychosis, or personality disorders.

^g^ Missing for 3597 MH patients and 16 420 PC without ambulatory or inpatient encounters in which to observe comorbidities during the 365 days prior to this visit.

### Firearm Question Response

In PC, 83.4% of patients answered the firearm access question; in MH, 91.8% of patients answered the question. In both PC and MH settings, nonresponse was statistically associated with older age, male sex, rural residence, and substance use disorder diagnoses (prior year) in adjusted and unadjusted analyses (Table 2 and Table 3). Nonresponse rates to the firearm access question (16.6% in PC; 8.2% in MH) were higher than the other questions on the MH monitoring questionnaire in both settings, including for alcohol consumption (2.5% in PC; 3.1% in MH), cannabis use (2.7% in PC; 2.6% in MH), and other drug use (3.4% for both settings) (eTable 1 in the Supplement).

**Table 2.** Response and Reported Access to a Standardized Question About Firearm Access in the Primary Care Setting—Observed Rates and Likelihood of Response Across Demographic and Clinical Characteristics at the Patient Level (Using the First Eligible Visit in the Study Period)^a^

Characteristic	Responded to the firearm question				Reported firearm access			
	Observed rates [n = 95 875], No. (%)		OR (95% CI)		Observed rates [n = 79 986], No. (%)		OR (95% CI)	
	No (16.6%)	Yes (83.4%)	Unadjusted (n = 95 875)	Adjusted (n = 79 215)^b^	No (79.1%)	Yes (20.9%)	Unadjusted (n = 79 986)	Adjusted (n = 66 313)^b^
Age category								
18-39 y	4473 (15.1)	25 161 (84.9)	1 [Reference]	1 [Reference]	20 991 (83.4)	4170 (16.6)	1 [Reference]	1 [Reference]
40-64 y	6719 (16.6)	33 875 (83.4)	0.90 (0.86-0.93)	0.92 (0.88-0.96)	25 883 (76.4)	7992 (23.6)	1.55 (1.49-1.62)	1.28 (1.21-1.34)
≥65 y	4679 (18.2)	20 950 (81.7)	0.79 (0.76-0.82)	0.81 (0.78-0.85)	16 421 (78.4)	4529 (21.6)	1.39 (1.32-1.45)	1.03 (0.97-1.09)
Sex								
Female	9516 (15.3)	52 702 (84.7)	1 [Reference]	1 [Reference]	44 026 (83.5)	8676 (16.5)	1 [Reference]	1 [Reference]
Male	6373 (18.9)	27 284 (81.1)	0.77 (0.75-0.80)	0.81 (0.78-0.85)	19 269 (70.6)	8015 (29.4)	2.11 (2.04-2.19)	2.10 (2.02-2.19)
Race and ethnicity								
American Indian/Alaska Native	255 (15.3)	1412 (84.7)	1.09 (0.95-1.25)	1.04 (0.09-1.21)	1095 (77.5)	317 (22.5)	0.97 (0.85-1.10)	1.07 (0.93-1.23)
Asian	981 (16.7)	4882 (83.3)	0.98 (0.91-1.05)	0.91 (0.84-0.98)	4486 (91.9)	396 (8.1)	0.30 (0.27-0.33)	0.41 (0.37-0.47)
Black	697 (17.9)	3194 (82.1)	0.90 (0.83-0.98)	0.81 (0.74-0.89)	2856 (89.4)	338 (10.6)	0.40 (0.35-0.44)	0.56 (0.49-0.63)
Hawaiian/Pacific Islander	147 (16.5)	744 (83.5)	1.00 (0.83-1.19)	0.90 (0.74-1.09)	629 (84.5)	115 (15.5)	0.61 (0.50-0.75)	0.71 (0.57-0.90)
Hispanic/Latinx	745 (16.4)	3796 (83.6)	1.00 (0.93-1.09)	0.92 (0.84-1.01)	3309 (87.2)	487 (12.8)	0.49 (0.45-0.54)	0.60 (0.54-0.67)
Other/unknown^c^	908 (17.6)	4265 (82.4)	0.93 (0.86-1.00)	0.87 (0.80-0.95)	3414 (80.0)	851 (20.0)	0.83 (0.77-0.90)	0.83 (0.76-0.91)
White	12 156 (16.5)	61 693 (83.5)	1 [Reference]	1 [Reference]	47 506 (77.0)	14 187 (23.0)	1 [Reference]	1 [Reference]
Insurance^d^								
Commercial	8455 (15.9)	44 739 (84.1)	1 [Reference]	NA	36 105 (80.7)	8634 (19.3)	1 [Reference]	NA
Medicare	4033 (18.2)	18 128 (81.8)	0.85 (0.82-0.89)	NA	14 537 (80.2)	3591 (19.8)	1.03 (0.99-1.08)	NA
Medicaid	767 (16.1)	3989 (83.9)	0.98 (0.91-1.07)	NA	3445 (86.4)	544 (13.6)	0.66 (0.60-0.73)	NA
Not enrolled	2634 (16.7)	13 130 (83.3)	0.94 (0.90-0.99)	NA	9208 (70.1)	3922 (29.9)	1.78 (1.70-1.86)	NA
Rural/urban^e^								
Urban	5222 (16.4)	26 565 (83.6)	1 [Reference]	1 [Reference]	23 784 (89.5)	2781 (10.5)	1 [Reference]	1 [Reference]
Suburban								
Large	3048 (14.4)	18 047 (85.6)	1.16 (1.11-1.22)	1.14 (1.09-1.20)	14 485 (80.3)	3562 (19.7)	2.10 (2.00-2.22)	2.12 (2.00-2.24)
Smaller	7085 (17.7)	32 990 (82.3)	0.92 (0.88-0.95)	0.89 (0.85-0.93)	23 498 (71.2)	9492 (28.8)	3.45 (3.30-3.62)	2.78 (2.64-2.93)
Mostly rural	489 (18.5)	2154 (81.5)	0.87 (0.78-0.96)	0.86 (0.77-0.97)	1345 (62.4)	809 (37.6)	5.14 (4.67-5.66)	4.25 (3.82-4.73)
Alcohol consumption (AUDIT-C)^f^								
Never	4444 (14.4)	26 431 (85.6)	1 [Reference]	NA	22 062 (83.5)	4369 (16.5)	1 [Reference]	NA
Low level	5696 (15.0)	32 375 (85.0)	0.96 (0.92-1.00)	NA	24 924 (77.0)	7451 (23.0)	1.51 (1.45-1.57)	NA
Moderate level	3656 (16.0)	19 182 (84.0)	0.88 (0.84-0.93)	NA	14 765 (77.0)	4417 (23.0)	1.51 (1.44-1.58)	NA
High level	355 (20.9)	1341 (79.1)	0.64 (0.56-0.72)	NA	1006 (75.0)	335 (25.0)	1.68 (1.48-1.91)	NA
Cannabis use^f^								
None	9926 (14.0)	61 074 (86.0)	1 [Reference]	NA	48 105 (78.8)	12 969 (21.2)	1 [Reference]	NA
Weekly-monthly	2260 (13.5)	14 450 (86.5)	1.04 (0.99-1.09)	NA	11 641 (80.6)	2809 (19.4)	0.90 (0.86-0.94)	NA
Daily/almost daily	1568 (28.4)	3962 (71.6)	0.41 (0.39-0.44)	NA	3165 (79.9)	797 (20.1)	0.93 (0.86-1.01)	NA
Other drug use^f^								
None	12 952 (14.3)	77 317 (85.7)	1 [Reference]	NA	61 096 (79.0)	16 221 (21.0)	1 [Reference]	NA
Daily-monthly	554 (24.0)	1756 (76.0)	0.53 (0.48-0.59)	NA	1465 (83.4)	291 (16.6)	0.75 (0.66-0.85)	NA
Depressive symptoms (PHQ-9)^f^								
None-minimal	7805 (17.0)	38 172 (83.0)	1 [Reference]	NA	29 129 (76.3)	9043 (23.7)	1 [Reference]	NA
Mild	3536 (15.8)	18 858 (84.2)	1.09 (1.04-1.14)	NA	15 073 (79.9)	3785 (20.1)	0.81 (0.78-0.84)	NA
Moderate	2148 (15.8)	11 467 (84.2)	1.09 (1.04-1.15)	NA	9414 (82.1)	2053 (17.9)	0.70 (0.67-0.74)	NA
Moderate-severe	1271 (15.6)	6853 (84.4)	1.10 (1.03-1.18)	NA	5702 (83.2)	1151 (16.8)	0.65 (0.61-0.70)	NA
Severe	773 (16.6)	3872 (83.4)	1.02 (0.94-1.11)	NA	3334 (86.1)	538 (13.9)	0.52 (0.47-0.57)	NA
Suicidal ideation (PHQ-9 question 9)^f^								
Not at all	13 724 (16.4)	69 996 (83.6)	1 [Reference]	NA	54 811 (78.3)	15 185 (21.7)	1 [Reference]	NA
Several days	1183 (16.1)	6182 (83.9)	1.02 (0.96-1.09)	NA	5266 (85.2)	916 (14.8)	0.63 (0.58-0.68)	NA
More than half	325 (16.3)	1665 (83.7)	1.00 (0.89-1.13)	NA	1416 (85.0)	249 (15.0)	0.63 (0.55-0.73)	NA
Nearly every day	199 (18.3)	888 (81.7)	0.87 (0.75-1.02)	NA	766 (86.3)	122 (13.7)	0.57 (0.47-0.70)	NA
Anxiety symptoms (GAD-2)^f^								
Negative	10 984 (16.5)	55 403 (83.5)	1 [Reference]	NA	42 781 (77.2)	12 622 (22.8)	1 [Reference]	NA
Positive	4439 (15.6)	24 099 (84.4)	1.08 (1.04-1.12)	NA	20 114 (83.5)	3985 (16.5)	0.67 (0.65-0.70)	NA
Depression diagnosis^g^								
No	10 145 (17.4)	48 266 (82.6)	1 [Reference]	1 [Reference]	36 871 (76.4)	11 395 (23.6)	1 [Reference]	1 [Reference]
Yes	5744 (15.3)	31 720 (84.7)	1.16 (1.12-1.20)	1.08 (1.03-1.12)	26 424 (83.3)	5296 (16.7)	0.65 (0.63-0.67)	0.86 (0.82-0.90)
Anxiety diagnosis^g^								
No	10 246 (17.5)	48 346 (82.5)	1 [Reference]	1 [Reference]	36 950 (76.4)	11 396 (23.6)	1 [Reference]	1 [Reference]
Yes	5643 (15.1)	31 640 (84.9)	1.19 (1.15-1.23)	1.10 (1.06-1.15)	26 345 (83.3)	5295 (16.7)	0.65 (0.63-0.68)	0.88 (0.84-0.92)
Serious mental illness diagnosis^g^								
No	15 466 (16.6)	77 941 (83.4)	1 [Reference]	1 [Reference]	61 486 (78.9)	16 455 (21.1)	1 [Reference]	1 [Reference]
Yes	423 (17.1)	2045 (82.9)	0.96 (0.86-1.07)	0.94 (0.84-1.06)	1809 (88.5)	236 (11.5)	0.49 (0.43-0.56)	0.56 (0.49-0.66)
Substance use disorder diagnosis^g^								
No	14 444 (16.3)	74 307 (83.7)	1 [Reference]	1 [Reference]	58 639 (78.9)	15 668 (21.1)	1 [Reference]	1 [Reference]
Yes	1445 (20.3)	5679 (79.7)	0.76 (0.72-0.81)	0.73 (0.69-0.78)	4656 (82.0)	1023 (18.0)	0.82 (0.77-0.88)	0.89 (0.82-0.96)
Suicide attempt diagnosis^g^								
No	15 847 (16.6)	79 829 (83.4)	1 [Reference]	1 [Reference]	63 148 (79.1)	16 681 (20.9)	1 [Reference]	1 [Reference]
Yes	42 (21.1)	157 (78.9)	0.74 (0.53-1.04)	0.77 (0.54-1.10)	147 (93.6)	10 (6.4)	0.26 (0.14-0.49)	0.38 (0.19-0.78)
Charlson Comorbidity Index score^h^								
0-1	10 760 (16.1)	56 006 (83.9)	1 [Reference]	1 [Reference]	45 105 (80.5)	10 901 (19.5)	1 [Reference]	1 [Reference]
≥2	2181 (17.2)	10 508 (82.8)	0.93 (0.88-1.07)	1.03 (0.97-1.08)	8285 (78.8)	2223 (21.2)	1.11 (1.05-1.17)	1.00 (0.95-1.06)

Abbreviations: AUDIT-C, Alcohol Use Disorders Identification Test Consumption; GAD-2, Generalized Anxiety Disorder 2-item; MH, mental health; NA, not applicable; OR, odds ratio; PC, primary care; PHQ-9, Patient Health Questionnaire.

^a^ Time-varying information defined at the time of the first outpatient visit during the study period. Includes information about 17 704 patients seen in both settings.

^b^ Adjusted for age, sex, race and ethnicity, rural/urban, diagnoses in year prior,^g^ Charlson score category,^h^ and prior-year enrollment category (not enrolled, <365 days, ≥356 days).

^c^ “Other” race and ethnicity was used to broadly capture all other write-in responses that did not fall into 1 of the broader categories, such as “Irish,” “Ashkenazi Jewish,” or “human race.”

^d^ Not enrolled insurance category includes patients of a Spokane-based health care system acquired by Kaiser Permanente Washington during the study period.

^e^ Based on a condensed version of the 2013 National Center for Health Statistics county urban-rural categorization. Missing for 275 patients.

^f^ Recorded via self-report on the MH monitoring questionnaire; see eTable 1 in the Supplement for a summary of nonresponse.

^g^ Diagnosis in year prior; serious mental illness diagnoses include bipolar, schizophrenia, other psychosis, or personality disorders.

^h^ Missing for 16 420 PC patients without ambulatory or inpatient encounters in which to observe comorbidities during the 365 days prior to this visit.

**Table 3.** Response and Reported Access to a Standardized Question About Firearm Access in the Outpatient Mental Health Specialty Setting—Observed Rates and Likelihood of Response Across Demographic and Clinical Characteristics at the Patient Level (Using the First Eligible Visit in the Study Period)^a^

Characteristic	Responded to the firearm question				Reported firearm access			
	Observed rates [n = 50 631], No. (%)		OR (95% CI)		Observed rates [n = 46 472], No. (%)		OR (95% CI)	
	No (8.2%)	Yes (91.8%)	Unadjusted (n = 50 631)	Adjusted (n = 46 772)^b^	No (84.6%)	Yes (15.3%)	Unadjusted (n = 46 472)	Adjusted (n = 42 917)^b^
Age category								
18-39 y	1875 (7.4)	23 306 (92.6)	1 [Reference]	NA	20 326 (87.2)	2980 (12.8)	1 [Reference]	1 [Reference]
40-64 y	1586 (8.6)	16 942 (91.4)	0.86 (0.80-0.92)	0.92 (0.85-0.99)	13 834 (81.7)	3108 (18.3)	1.53 (1.45-1.62)	1.37 (1.29-1.45)
≥65 y	698 (10.1)	6224 (89.9)	0.72 (0.65-0.79)	0.82 (0.73-0.91)	5178 (83.2)	1046 (16.8)	1.38 (1.28-1.49)	1.12 (1.02-1.22)
Sex								
Female	2547 (8.0)	29 424 (92.0)	1 [Reference]	1 [Reference]	25 629 (87.1)	3795 (12.9)	1 [Reference]	1 [Reference]
Male	1612 (8.6)	17 047 (91.4)	0.92 (0.86-0.98)	0.92 (0.86-0.99)	13 708 (80.4)	3339 (19.6)	1.64 (1.56-1.73)	1.76 (1.67-1.86)
Race and ethnicity								
American Indian/Alaska Native	90 (8.4)	985 (91.6)	0.99 (0.80-1.24)	0.99 (0.79-1.24)	807 (81.9)	178 (18.1)	1.10 (0.94-1.30)	1.08 (0.91-1.29)
Asian	204 (7.4)	2541 (92.6)	1.13 (0.98-1.31)	0.97 (0.83-1.14)	2324 (91.5)	217 (8.5)	0.47 (0.40-0.54)	0.59 (0.51-0.69)
Black	173 (7.4)	2165 (92.6)	1.14 (0.97-1.33)	1.00 (0.84-1.18)	1971 (91.0)	194 (9.0)	0.49 (0.42-0.57)	0.56 (0.48-0.66)
Hawaiian/Pacific Islander	46 (8.7)	485 (91.3)	0.96 (0.71-1.30)	0.90 (0.65-1.25)	424 (87.4)	61 (12.6)	0.72 (0.55-0.94)	0.81 (0.61-1.07)
Hispanic/Latinx	225 (8.1)	2554 (91.9)	1.03 (0.90-1.19)	0.93 (0.80-1.08)	2271 (88.9)	283 (11.1)	0.62 (0.55-0.71)	0.71 (0.62-0.81)
Other/unknown^c^	227 (7.9)	2628 (92.1)	1.05 (0.92-1.21)	1.03 (0.88-1.20)	2281 (86.8)	347 (13.2)	0.76 (0.68-0.85)	0.81 (0.71-0.92)
White	3194 (8.3)	35 114 (91.7)	1 [Reference]	1 [Reference]	29 260 (83.3)	5854 (16.7)	1 [Reference]	1 [Reference]
Insurance^d^								
Commercial	2797 (7.6)	33 946 (92.4)	1 [Reference]	NA	28 525 (84.0)	5421 (16.0)	1 [Reference]	NA
Medicare	948 (10.6)	8017 (89.4)	0.70 (0.64-0.75)	NA	6731 (84.0)	1286 (16.0)	1.01 (0.94-1.07)	NA
Medicaid	250 (8.0)	2876 (92.0)	0.96 (0.83-1.08)	NA	2580 (89.7)	296 (10.3)	0.60 (0.53-0.68)	NA
Not enrolled	164 (9.1)	1633 (90.9)	0.82 (0.70-0.97)	NA	1502 (92.0)	131 (8.0)	0.46 (0.38-0.55)	NA
Rural/urban^e^								
Urban	1517 (7.2)	19 694 (92.8)	1 [Reference]	1 [Reference]	17 943 (91.1)	1751 (8.9)	1 [Reference]	1 [Reference]
Suburban								
Large	801 (6.1)	12 296 (93.9)	1.18 (1.08-1.29)	1.19 (1.08-1.31)	10 133 (82.4)	2163 (17.6)	2.19 (2.04-2.34)	2.22 (2.06-2.38)
Smaller	1518 (10.6)	12 792 (89.4)	0.65 (0.60-0.70)	0.64 (0.59-0.69)	10 033 (78.4)	2759 (21.6)	2.82 (2.64-3.01)	2.77 (2.58-2.96)
Mostly rural	306 (17.6)	1434 (82.4)	0.36 (0.32-0.41)	0.37 (0.32-0.43)	1008 (70.3)	426 (29.7)	4.33 (3.83-4.99)	4.14 (3.64-4.72)
Alcohol consumption (AUDIT-C)^f^								
Never	1160 (7.5)	14 213 (92.5)	1 [Reference]	NA	12 442 (87.5)	1771 (12.5)	1 [Reference]	NA
Low level	1243 (6.3)	18 386 (93.7)	1.21 (1.11-1.31)	NA	15 232 (82.8)	3154 (17.2)	1.45 (1.37-1.55)	NA
Moderate level	806 (6.3)	12 001 (93.7)	1.22 (1.11-1.33)	NA	10 103 (84.2)	1898 (15.8)	1.32 (1.23-1.42)	NA
High level	89 (7.2)	1146 (92.8)	1.05 (0.84-1.31)	NA	941 (82.1)	205 (17.9)	1.53 (1.31-1.79)	NA
Cannabis use^f^								
None	1929 (6.0)	30 138 (94.0)	1 [Reference]	NA	25 396 (84.3)	4742 (15.7)	1 [Reference]	NA
Weekly-monthly	691 (5.8)	11 307 (94.2)	1.05 (0.96-1.15)	NA	9722 (86.0)	1585 (14.0)	0.87 (0.82-0.93)	NA
Daily/almost daily	565 (10.8)	4666 (89.2)	0.53 (0.48-0.58)	NA	3914 (83.9)	752 (16.1)	1.03 (0.95-1.12)	NA
Other drug use^f^								
None	2830 (6.1)	43 259 (93.9)	1 [Reference]	NA	36 554 (84.5)	6705 (15.5)	1 [Reference]	NA
Daily-monthly	271 (9.7)	2525 (90.3)	0.61 (0.53-0.69)	NA	2187 (86.6)	338 (13.4)	0.84 (0.75-0.95)	NA
Depressive symptoms (PHQ-9)^f^								
None-minimal	944 (8.7)	9901 (91.3)	1 [Reference]	NA	8373 (84.6)	1528 (15.4)	1 [Reference]	NA
Mild	1043 (7.9)	12 103 (92.1)	1.11 (1.01-1.21)	NA	10 163 (84.0)	1940 (16.0)	1.05 (0.97-1.13)	NA
Moderate	926 (8.2)	10 347 (91.8)	1.07 (0.97-1.17)	NA	8760 (84.7)	1587 (15.3)	0.99 (0.92-1.07)	NA
Moderate-severe	695 (8.1)	7900 (91.9)	1.08 (0.98-1.20)	NA	6649 (84.2)	1251 (15.8)	1.03 (0.95-1.12)	NA
Severe	527 (8.0)	6089 (92.0)	1.10 (0.99-1.23)	NA	5272 (86.6)	817 (13.4)	0.85 (0.77-0.93)	NA
Suicidal ideation (PHQ-9 question 9)^f^								
Not at all	3035 (8.1)	34 562 (91.9)	1 [Reference]	NA	28 984 (83.9)	5578 (16.1)	1 [Reference]	NA
Several days	644 (8.1)	7304 (91.9)	1.00 (0.91-1.09)	NA	6321 (86.5)	983 (13.5)	0.81 (0.75-0.87)	NA
More than half	255 (9.1)	2536 (90.9)	0.87 (0.76-1.00)	NA	2200 (86.8)	336 (13.2)	0.79 (0.71-0.89)	NA
Nearly every day	191 (9.3)	1866 (90.7)	0.86 (0.74-1.00)	NA	1651 (88.5)	215 (11.5)	0.68 (0.59-0.78)	NA
Anxiety symptoms (GAD-2)^f^								
Negative	1874 (8.8)	19 447 (91.2)	1 [Reference]	NA	16 368 (84.2)	3079 (15.8)	1 [Reference]	NA
Positive	2255 (7.8)	26 828 (92.2)	1.15 (1.08-1.22)	NA	22 802 (85.0)	4026 (15.0)	0.94 (0.89-0.99)	NA
Depression diagnosis^g^								
No	1587 (8.5)	16 976 (91.5)	1 [Reference]	1 [Reference]	14 278 (84.1)	2698 (15.9)	1 [Reference]	1 [Reference]
Yes	2572 (8.0)	29 496 (92.0)	1.07 (1.00-1.14)	1.01 (0.94-1.08)	25 060 (85.0)	4436 (15.0)	0.94 (0.89-0.99)	0.87 (0.82-0.92)
Anxiety diagnosis^g^								
No	1082 (9.4)	10 457 (90.6)	1 [Reference]	1 [Reference]	8791 (84.1)	1666 (15.9)	1 [Reference]	1 [Reference]
Yes	3077 (7.9)	36 015 (92.1)	1.21 (1.13-1.30)	1.18 (1.09-1.28)	30 547 (84.8)	5468 (15.2)	0.93 (0.89-1.00)	0.95 (0.89-1.02)
Serious mental illness diagnosis^g^								
No	3422 (7.8)	40 315 (92.2)	1 [Reference]	1 [Reference]	33 827 (83.9)	6488 (16.1)	1 [Reference]	1 [Reference]
Yes	737 (10.7)	6157 (89.3)	0.71 (0.65-0.77)	0.73 (0.66-0.80)	5511 (89.5)	646 (10.5)	0.61 (0.56-0.67)	0.56 (0.51-0.61)
Substance use disorder diagnosis^g^								
No	3450 (8.0)	39 843 (92.0)	1 [Reference]	1 [Reference]	33 672 (84.5)	6171 (15.5)	1 [Reference]	1 [Reference]
Yes	709 (9.7)	6629 (90.3)	0.81 (0.74-0.88)	0.84 (0.76-0.92)	5666 (85.5)	963 (14.5)	0.93 (0.86-1.00)	0.90 (0.83-0.98)
Suicide attempt diagnosis^g^								
No	4116 (8.2)	45 959 (91.8)	1 [Reference]	1 [Reference]	38 849 (84.5)	7110 (15.5)	1 [Reference]	1 [Reference]
Yes	43 (7.7)	513 (92.3)	1.07 (0.78-1.46)	1.20 (0.87-1.66)	489 (95.3)	24 (4.7)	0.27 (0.18-0.40)	0.31 (0.20-0.47)
Charlson Comorbidity Index score^h^								
0-1	3313 (8.0)	38 223 (92.0)	1 [Reference]	1 [Reference]	32 369 (84.7)	5854 (15.3)	1 [Reference]	1 [Reference]
≥2	558 (10.2)	4940 (91.8)	0.77 (0.70-0.84)	0.91 (0.82-1.01)	4034 (81.7)	906 (18.3)	1.24 (1.15-1.34)	1.07 (0.98-1.17)

Abbreviations: AUDIT-C, Alcohol Use Disorders Identification Test Consumption; GAD-2, Generalized Anxiety Disorder 2-item; MH, mental health; OR, odds ratio; PHQ-9, Patient Health Questionnaire.

^a^ Time-varying information defined at the time of the first outpatient visit during the study period. Includes information about 17 704 patients seen in both settings.

^b^ Adjusted for age, sex, race and ethnicity, rural/urban, diagnoses in year prior,^g^ Charlson score category,^h^ and prior year enrollment category (not enrolled, <365 days, ≥356 days).

^c^ “Other” race and ethnicity was used to broadly capture all other write-in responses that did not fall into 1 of the broader categories, such as “Irish,” “Ashkenazi Jewish,” or “human race.”

^d^ Sex missing for 1 patient; the “not enrolled” insurance category includes patients of a Spokane-based health care system acquired by Kaiser Permanente Washington during the study period.

^e^ Based on a condensed version of the 2013 National Center for Health Statistics county urban-rural categorization. Missing for 273 patients.

^f^ Recorded via self-report on the MH monitoring questionnaire; see eTable 1 in the Supplement for a summary of nonresponse.

^g^ Diagnosis in year prior; serious mental illness diagnoses include bipolar, schizophrenia, other psychosis, or personality disorders.

^h^ Missing for 3597 patients without ambulatory or inpatient encounters in which to observe comorbidities during 365 days prior to this visit.

### Firearm Access Reported

In PC, 20.9% of patients who responded to the firearm question reported having access; in MH, 15.3% of patients reported having access. Most sociodemographic and clinical characteristics were statistically associated with reporting firearm access, but the magnitude of differences was largest (±10%) for sex, rural/urban residence, and prior-year suicide attempt diagnosis (Table 2). In PC, men were more likely than women to report access (16.5% vs 29.4%; adjusted OR = 2.10; 95% CI, 2.02-2.19); those in mostly rural areas compared with urban areas were more likely to report access (10.5% vs 37.6%; adjusted OR = 4.25; 95% CI, 3.82-4.73); and patients with a prior-year suicide attempt diagnosis were less likely than those with no diagnosis to report access (6.4% vs 20.9%; adjusted OR = 0.38; 95% CI, 0.19-0.78). Similar characteristics were associated with reporting firearm access in MH, and the magnitude of the differences was largest (±10%) for rural/urban residence and prior-year suicide attempt diagnosis (Table 3).

### Sensitivity Analyses

Results using all eligible visits in the study period were similar to main analyses (eTables 2 and 3 in the Supplement).

### Response and Access Consistency

**Figure 2.**
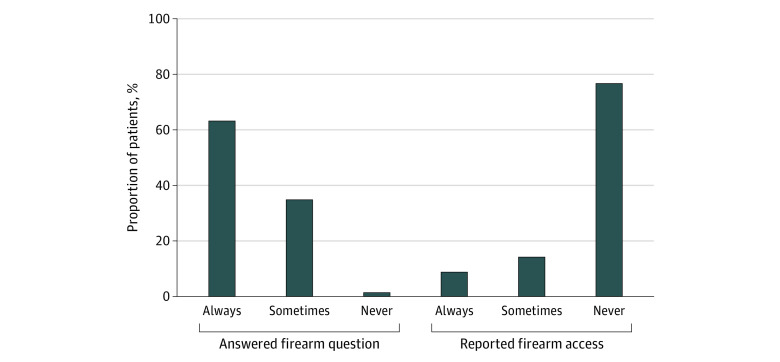
Responses to the Firearm Access Question Among Patients With More Than 1 Visit to an Outpatient Mental Health and/or Primary Care Clinician During the Study Period Proportions of patients with 2 or more visits during the study period (n = 60 514) who always, sometimes, or never (1) answered the firearm access question and (2) among those who answered at least twice (n = 54 915), always, sometimes, or never reported firearm access.

About half (60 514 [47.0%]) the patients in the sample had 2 or more eligible visits; 63.3% always answered, 35.0% sometimes answered, and 1.7% never answered the firearm access question. Of patients in this sample who answered at least twice (n = 54 915), 9.0% always reported firearm access, 14.2% sometimes reported firearm access, and 76.8% never reported access (Figure 2). The proportion of patients with 2 or more eligible visits who sometimes answered the firearm access question and the proportion who sometimes reported access increased with greater number of visits and longer intervals between the first and last visit (eTable 4 in the Supplement).

## Discussion

In this cross-sectional study of adult patients receiving care for MH and substance use, 83.4% of PC patients answered a question about firearm access, and 20.9% of patients who responded reported access. In MH, 91.8% of patients answered the question, and 15.3% reported access. Examination of the correlates of reported firearm access indicated that the prevalence in PC and MH was highest among men, White patients, and those living in smaller suburban and mostly rural areas. Prevalence of patient-reported firearm access was lowest for younger adults, patients living in urban areas, and those reporting greater severity of depressive symptoms, more frequent suicidal ideation, and those with a past-year diagnosis of depression, anxiety, serious mental illness, substance use disorder, or a prior-year suicide attempt.

This novel study demonstrates that standard assessment of access to firearms is feasible in the context of MH monitoring in PC and outpatient MH specialty settings. This finding extends prior population-based survey research indicating that most patients believe that questions about firearm access are appropriate for suicide prevention^40^ and qualitative research describing how adult patients perceived a standard question about firearm access as being relevant to their MH care.^41^ The rates and correlates of reported firearm access in this large health care system population are also consistent with prior research in Washington State^25,42,43,44^ demonstrating higher rates of firearm access among men and adult respondents who were older, White, living in rural areas, and reporting higher levels of alcohol consumption. Findings are also consistent with survey-based studies among adult clinical populations reporting lower rates of firearm access among those also reporting prior suicide attempts^45,46^ or suicidal thoughts.^45,46,47^ In contrast, indicators of depression, anxiety, serious mental illnesses, and substance use disorders have not consistently been associated with firearm access in prior studies.^25,45,46,47,48^

Health systems that routinely collect standard patient-reported firearm access data on population-based questionnaires can use this information to guide applicable follow-up care. Asking patients to routinely self-report firearm access can help clinicians identify and engage patients at risk of suicide in dialogue regarding storage of firearms and/or ammunition (ie, increasing time and/or distance required to access firearms), which is a recommended component of evidence-based safety planning interventions for suicide prevention.^14,17,18^ Yet the quality of the safety planning practices is variable,^15,49,50^ and using information reported in response to standard questions about firearm access, instead of relying on clinicians to decide whether to ask patients, may help improve this practice. However, despite potential benefits, national debate remains as to whether and how health care organizations should collect and store firearm access information.^51,52,53,54^ No federal law or statute prohibits clinicians from asking about firearms when the information is relevant to patients’ health,^55^ but there remains a dearth of national recommendations/guidelines for implementing firearm assessment and follow-up in clinical practice.^4^ Efforts to increase assessment must be paired with information on how to follow up. Therefore, interdisciplinary groups of clinicians and public health experts have developed clinician-facing resources to support dialogue with patients about firearm risk and safety,^56^ as well as patient-facing tools, such as a web-based decision aid developed in collaboration with firearm owners and individuals with suicidality.^57,58^ Responses to standard firearm access questions used in combination with resources designed to help patients at risk of suicide make safe decisions about firearm access and storage may improve suicide prevention practices and outcomes.^23,59^

### Limitations

This cross-sectional study has important limitations. First, only in-person visits were included, because virtual visits were rare at the time of this analysis. Additional research is needed to explore virtual patient-reported firearm access.^60^ Second, it is not possible to know from this analysis whether or how response is associated with access; patients with reported access to firearms may have been less likely to respond to the question, as nonresponse was associated with characteristics also associated with higher reported access (eg, older age, male sex, rural residence). Third, additional analyses are needed to understand what patient characteristics are associated with changes in reported firearm access between visits. Fourth, patients were not geographically representative of Washington state and results may not be generalizable to rural and noninsured clinical populations. Moreover, the firearm question was routinely administered to PC patients with a prior MH or substance use disorder diagnosis; sociodemographic and clinical characteristics likely influence whether patients receive these diagnoses.^61,62^ Finally, concerns about privacy, surveillance, or Extreme Risk Protection Orders (“red flag” laws) in many states (including Washington)^63,64^ may affect how patients perceive and answer firearm questions (ie, reported access may not reflect “true state”).^41^ Though response rates were high in PC and MH settings, nonresponse rates were higher to this question than to all the other questions, which supports prior qualitative findings indicating that practices designed to clarify the purpose and use of firearm access may encourage firearm access disclosures and facilitate dialogue about safe storage.^41^

## Conclusions

Findings from this novel cross-sectional study among patients receiving care for MH and/or substance use in PC or outpatient MH specialty clinics provide a critical foundation to help advance our understanding of the utility of standardized firearm access assessment practices. This research demonstrated that including a standard question about firearm access on an MH monitoring questionnaire was feasible; patients answered and reported access. Future work should focus on improving the patient-centeredness and effectiveness of this practice, used in combination with resources designed to support dialogue and mindful decision-making about firearm access.

## Notes

Supplement. **eFigure 1.** Mental Health Monitoring Questionnaire **eFigure 2.** EHR-Based Reminders Designed to Prompt Use of the Mental Health Monitoring Questionnaire **eAppendix.** Analytic Dataset Specifications **eTable 1.** Summary of non-response in the PC and MH settings (1/1/2016-12/31/2019), at the patient and visits levels (using first visit and all visits in the study period) **eTable 2.** Response and reported access to a standardized question about firearm access in the primary care setting, observed rates and likelihood of response across demographic and clinical characteristics at the visit-level (using all visits in the study period) **eTable 3.** Response and reported access to a standardized question about firearm access in the outpatient mental health specialty setting, observed rates and likelihood of response across demographic and clinical characteristics at the visit-level (using all visits in the study period) **eTable 4.** Consistency of patient-reported firearm access among patients with two or more visits during the study period, stratified by the number of visits and interval between the first and last visit during the study period
