# Error in Funding/Support

**DOI:** 10.1001/jamahealthforum.2021.2986

**Published:** 2021-09-17

The Original Investigation titled, “Self-reported Access to Firearms Among Patients Receiving Care for Mental Health and Substance Use,”^1^ published online August 6, 2021, included an error in the Funding/Support section. The work was funded by Kaiser Permanente’s Office of Community Health as part of its Firearm Injury Prevention Program. The article has been corrected.
