# Supplementary material for: Self-reported Access to Firearms Among Patients Receiving Care for Mental Health and Substance Use
Source: JAMA Health Forum. 2021 Aug 6;2(8):e211973. doi: 10.1001/jamahealthforum.2021.1973 (PMC8796974; doi:10.1001/jamahealthforum.2021.1973)
Supplement: Supplement. — eFigure 1. Mental Health Monitoring Questionnaire eFigure 2. EHR-Based Reminders Designed to Prompt Use of the Mental Health Monitoring Questionnaire eAppendix. Analytic Dataset Specifications eTable 1. Summary of non-response in the PC and MH settings (1/1/2016-12/31/2019), at the patient and visits levels (using first visit and all visits in the study period) eTable 2. Response and reported access to a standardized question about firearm access in the primary care setting, observed rates and likelihood of response across demographic and clinical characteristics at the visit-level (using all visits in the study period) eTable 3. Response and reported access to a standardized question about firearm access in the outpatient mental health specialty setting, observed rates and likelihood of response across demographic and clinical characteristics at the visit-level (using all visits in the study period) eTable 4. Consistency of patient-reported firearm access among patients with two or more visits during the study period, stratified by the number of visits and interval between the first and last visit during the study period [file jamahealthforum-e211973-s001.pdf]

Supplemental Online Content

Richards JE, Kuo E, Stewart C, et al. Self-reported access to firearms among patients receiving care for mental health and substance use. *JAMA Health Forum*. 2021;2(8):e211973. doi:10.1001/jamahealthforum.2021.1973

eFigure 1. Mental Health Monitoring Questionnaire

eFigure 2. EHR-Based Reminders Designed to Prompt Use of the Mental Health Monitoring Questionnaire

eAppendix. Analytic Dataset Specifications

eTable 1. Summary of non-response in the PC and MH settings (1/1/2016-12/31/2019), at the patient and visits levels (using first visit and all visits in the study period)

eTable 2. Response and reported access to a standardized question about firearm access in the primary care setting, observed rates and likelihood of response across demographic and clinical characteristics at the visit-level (using all visits in the study period)

eTable 3. Response and reported access to a standardized question about firearm access in the outpatient mental health specialty setting, observed rates and likelihood of response across demographic and clinical characteristics at the visit-level (using all visits in the study period)

eTable 4. Consistency of patient-reported firearm access among patients with two or more visits during the study period, stratified by the number of visits and interval between the first and last visit during the study period

This supplemental material has been provided by the authors to give readers additional information about their work.

© 2021 Richards JE et al. *JAMA Health Forum.*

eFigure 1. Mental Health Monitoring Questionnaire

Patient Label

Name: ____________________

MRN: ____________________

Date: ____________________

KAISER PERMANENTE.

Mental Health Monitoring Tool

Over the past 2 weeks, how often have you been bothered by any of the following problems?	Not at all	Several days	More than half the days	Nearly every day
1. Little interest or pleasure in doing things	0	1	2	3
2. Feeling down, depressed or hopeless	0	1	2	3
3. Trouble falling or staying asleep or sleeping too much	0	1	2	3
4. Feeling tired or having little energy	0	1	2	3
5. Poor appetite or overeating	0	1	2	3
6. Feeling bad about yourself – or that you are a failure or have let yourself or family down	0	1	2	3
7. Trouble concentrating on things, such as reading the newspaper or watching television	0	1	2	3
8. Moving or speaking so slowly that other people could have noticed. Or the opposite – being so fidgety or restless thatyou have been moving around a lot more than usual.	0	1	2	3
9. Thoughts that you would be better off dead or of hurting yourself in some way	0	1	2	3
10. Feeling nervous, anxious or on edge	0	1	2	3
11. Not being able to stop or control worrying	0	1	2	3
12. Have your problems interfered with your work, family or social activities?	0	1	2	3

Please answer these questions about the past year. (If you have changed your drinking or substance use in the past year, please report on your most recent use.)

Question						
13. How often do you have a drink containing alcohol?	Never 0	Monthly or less 1	2 to 4 times a month 2		2 to 3 times a week 3	4 or more times a week 4
14. How many drinks containing alcohol do you have on a typical day when you are drinking?	None 0	1 or 2 drinks 0	3 or 4 drinks 1	5 or 6 drinks 2	7 to 9 drinks 3	10 or more drinks 4
15. How often do you have 6 or more drinks on one occasion?	Never 0	Less than monthly 1	Monthly 2		Weekly 3	Daily or almost daily 4
16. How often have you used marijuana?	Never 0	Less than monthly 1	Monthly 2		Weekly 3	Daily or almost daily 4
17. How often have you used an illegal drug (not marijuana) or used a prescription medication for non-medical reasons?	Never 0	Less than monthly 1	Monthly 2		Weekly 3	Daily or almost daily 4
18. Do you have access to guns?			Yes	No		

© 2021 Richards JE et al. *JAMA Health Forum.*

eFigure 2. EHR-Based Reminders Designed to Prompt Use of the Mental Health Monitoring Questionnaire

<u>Depression</u>

On Medication:

- Assess with MH Monitoring Tool. <u>Provider</u>: Patient has active Major Depression on antidepressant treatment ([Last Diagnosis]). HEDIS measure for patient ≥ 18 years old in initial treatment period: Continue antidepressant through [DATE] (12 weeks). Review monitoring tool and adjust treatment based on assessment.

- Assess with MH Monitoring Tool. Provider: Patient has active Major Depression on antidepressant treatment ([Last Diagnosis]). HEDIS measure for patient ≥ 18 years old in continued treatment period: Continue antidepressant through [DATE] (6 months). Review monitoring tool and adjust treatment based on assessment.

Not on Medication:

- Assess with MH Monitoring Tool. <u>Provider</u>: Patient has active diagnosis for depression ([Last Diagnosis]), not on medication. Review monitoring tool and adjust treatment based on assessment.

- Assess with MH Monitoring Tool. <u>Provider</u>: Patient has active diagnosis for depression ([Last Diagnosis]), not on medication. Review monitoring tool and adjust treatment based on assessment.

<u>Alcohol and Drug Use Disorder</u>

- Assess with MH Monitoring Tool. <u>Provider</u>: Patient has active alcohol or drug use disorder [Last Diagnosis]. HEDIS measure for patients ≥ 18 years old: need 2 more follow-up visits by [DATE]. If you will not address and code today, please contact SW.

- Assess with MH Monitoring Tool. <u>Provider</u>: Patient has active alcohol or drug use disorder [Last Diagnosis]. HEDIS measure for patients ≥ 18 years old: need 1 more follow-up visit by [DATE]. If you will not address and code today, please contact SW.

<u>Missed Assessment Prompt</u>

Used when patients screened positive for depression on the PHQ-2 but did not complete the full PHQ-9 at a prior visit (within the last year).

- Due for BHI Monitoring Tool or PHQ-9.

© 2021 Richards JE et al. *JAMA Health Forum.*

eAppendix. Analytic Dataset Specifications

Below is a description of the general approach used to assemble the dataset used to analyze patient-reported firearm access among adults receiving mental health care.

Step 1: Pull from EPIC Clarity database all mental health monitoring questionnaires 1/1/2016-12/31/2019, with associated encounter types/dates, departments, and provider types. Assign a unique ID to each mental health monitoring questionnaire and unique person ID (individuals may have multiple monitoring tools).

Step 2: Link sociodemographic and clinical variables, defined using KPWHRI VDW and MHRN variable definitions, to each mental health monitoring tool. Details available here: https://github.com/MHResearchNetwork/Diagnosis-Codes

Step 3: Exclude patients < 18 years old, and individuals on the no research list.

Preparatory Analyses:

- Review all encounter types associated with administration of the monitoring tool.
- Review all departments associated with administration of the monitoring tool.
- Categorize departments where the monitoring tool is used by care setting (e.g. primary care, mental health specialty, other). Primary care will include family practice, general internal medicine, pediatrics, women's health, and social work (i.e. due to introduction of integrated mental health social workers in this setting).
- Calculate distribution of monitoring tools administered by setting by year; confirm patterns correspond with implementation of integrated mental health in primary care.
- Examine the distribution of mental health monitoring tools completed by individual patients during the study period by care setting.
- Examine patterns of missing data for each covariate by care setting (additionally explore by year as needed).

Non-response to firearm access question: Based on preliminary analyses and consultation with clinical leaders, the presence of the GAD-2 questions on the monitoring tool, without a matching response on the firearm question will be used to define non-response. The GAD-2 will be used for this purpose because it is specific to the monitoring tool, and because patients are more likely to answer the GAD-2 (based on clinical experience) than substance use questions (these are also often left blank).

© 2021 Richards JE et al. *JAMA Health Forum.*

eTable 1. Summary of non-response in the PC and MH settings (1/1/2016-12/31/2019), at the patient and visits levels (using first visit and all visits in the study period)

	PC Setting				MH Setting			
	First Visit N=95875		All Visits N=160856		First Visit N=50631		All Visits N=327632	
	N	%	N	%	N	%	N	%
Alcohol Consumption (AUDIT-C)	2395	2.5%	4965	3.1%	1587	3.1%	17186	5.3%
Cannabis Use	2635	2.7%	5262	3.3%	1335	2.6%	15578	4.7%
Other Drug Use	3296	3.4%	6501	4.0%	1746	3.4%	18084	5.5%
Depressive Symptoms (PHQ-9‡)	1120	1.2%	1539	1.0%	156	0.3%	1168	0.4%
Suicidal ideation (PHQ-9 Q9)	1713	1.8%	2591	1.6%	238	0.5%	1626	0.5%
Anxiety Symptoms (GAD-2†)	950	1.0%	1437	0.9%	227	0.4%	1137	0.4%
Access to Firearms	15889	16.6%	25675	16.0%	4159	8.2%	36867	11.3%

†The presence of a response to either of GAD-2 questions were used to define responses to the question about access to firearms, because this question was unique to the mental health monitoring questionnaire during the study period.

‡Up to 2 missing values were allowed for the PHQ-9; total score was completed using the average score of the completed items.

© 2021 Richards JE et al. *JAMA Health Forum.*

eTable 2. Response and reported access to a standardized question about firearm access in the primary care setting, observed rates and likelihood of response across demographic and clinical characteristics at the visit-level (using all visits in the study period)

	Responded to the firearm question?								Reported firearm access?							
	Observed Rates N=160,856				Unadjusted N=160,856		Adjusted‡‡ N=141,064		Observed Rates N=135,181				Unadjusted N=135,181		Adjusted‡‡ N=118,927	
	No (16.0%)		Yes (84.0%)		OR	(95% CI)	OR	(95% CI)	No (80.7%)		Yes (19.4%)		OR	95% CI	OR	95% CI
**Age Category**																
18-39	7172	14.5%	42460	85.5%	Ref		Ref		35989	84.8%	6471	15.2%	Ref		Ref	
40-64	10783	16.1%	56117	83.9%	0.88	(0.85-0.91)	0.90	(0.86-0.94)	43634	77.8%	12483	22.2%	1.59	(1.51-1.67)	1.33	(1.25-1.41)
65+	7720	17.4%	36604	82.6%	0.80	(0.77-0.83)	0.81	(0.77-0.85)	29402	80.3%	7202	19.7%	1.36	(1.29-1.44)	1.07	(1.00-1.15)
**Sex**																
Female	16298	15.0%	92469	85.0%	Ref		Ref		77941	84.3%	14528	15.7%	Ref		Ref	
Male	9377	18.0%	42712	82.0%	0.80	(0.78-0.83)	0.85	(0.82-0.88)	31084	72.8%	11628	27.2%	2.01	(1.92-2.10)	2.00	(1.91-2.11)
**Race and Ethnicity**																
American Indian/ Alaska Native	477	14.8%	2752	85.2%	1.08	(0.96-1.22)	1.03	(0.91-1.16)	2206	80.2%	546	19.8%	0.92	(0.78-1.08)	0.98	(0.82-1.17)
Asian	1438	15.9%	7593	84.1%	0.99	(0.93-1.06)	0.90	(0.84-0.97)	6948	91.5%	645	8.5%	0.35	(0.30-0.39)	0.46	(0.40-0.53)
Black	1235	17.4%	5867	82.6%	0.89	(0.83-0.96)	0.80	(0.74-0.87)	5247	89.4%	620	10.6%	0.44	(0.39-0.50)	0.61	(0.53-0.70)
Hawaiian/Pacific Islander	243	16.5%	1232	83.5%	0.95	(0.80-1.13)	0.85	(0.71-1.03)	1055	85.6%	177	14.4%	0.62	(0.49-0.79)	0.73	(0.56-0.95)
Hispanic/Latinx	1311	15.9%	6922	84.1%	0.99	(0.92-1.07)	0.89	(0.83-0.97)	6085	87.9%	837	12.1%	0.51	(0.45-0.58)	0.62	(0.53-0.72)
other/unk**	1353	17.3%	6492	82.8%	0.90	(0.84-0.97)	0.85	(0.78-0.92)	5272	81.2%	1220	18.8%	0.86	(0.79-0.94)	0.84	(0.76-0.94)
white	19618	15.8%	104323	84.2%	Ref		Ref		82212	78.8%	22111	21.2%	Ref		Ref	
**Insurance***																
Commercial	13537	15.2%	75683	84.8%	Ref				61616	81.4%	14067	18.6%	Ref			
Medicare	7449	17.3%	35570	82.7%	0.85	(0.82-0.89)			28984	81.5%	6586	18.5%	1.00	(0.94-1.05)		
Medicaid	1519	15.8%	8067	84.2%	0.95	(0.89-1.02)			6986	86.6%	1081	13.4%	0.68	(0.60-0.76)		
Not Enrolled	3170	16.7%	15861	83.3%	0.89	(0.86-0.94)			11439	72.1%	4422	27.9%	1.69	(1.61-1.78)		
**Rural/Urban*** **																
Urban	8630	15.6%	46766	84.4%	Ref		Ref		42004	89.8%	4762	10.2%	Ref		Ref	
Large Suburban	5960	13.9%	36787	86.1%	1.14	(1.09-1.19)	1.11	(1.06-1.16)	29711	80.8%	7076	19.2%	2.10	(1.97-2.24)	2.13	(1.99-2.78)
Smaller Suburban	10243	17.7%	47755	82.3%	0.86	(0.83-0.89)	0.85	(0.81-0.88)	34759	72.8%	12996	27.2%	3.30	(3.12-3.49)	2.78	(2.60-2.97)
Mostly Rural	774	18.2%	3475	81.8%	0.83	(0.75-0.91)	0.84	(0.76-0.93)	2250	64.7%	1225	35.3%	4.80	(4.29-5.37)	4.18	(3.69-4.72)
**Alcohol Consumption† (AUDIT-C)**																
Never	7452	13.4%	48253	86.6%	Ref				40540	84.0%	7713	16.0%	Ref			
Low-Level	8602	13.8%	53744	86.2%	0.96	(0.93-1.00)			42368	78.8%	11376	21.2%	1.41	(1.35-1.48)		
Moderate-Level	5251	15.0%	29816	85.0%	0.88	(0.84-0.91)			23471	78.7%	6345	21.3%	1.42	(1.35-1.50)		
High-Level	550	19.8%	2223	80.2%	0.62	(0.56-0.69)			1707	76.8%	516	23.2%	1.59	(1.39-1.81)		
**Cannabis Use†**																
None	14925	12.8%	101739	87.2%	Ref				81854	80.5%	19885	19.5%	Red			
Weekly-Monthly	3626	12.6%	25104	87.4%	1.02	(0.97-1.06)			20514	81.7%	4590	18.3%	0.92	(0.88-0.97)		
Daily/Almost Daily	2681	26.3%	7519	73.7%	0.41	(0.39-0.43)			6011	79.9%	1508	20.1%	1.03	(0.94-1.13)		
**Other Drug Use†**																
None	19902	13.2%	130555	86.8%	Ref				105200	80.6%	25355	19.4%	Ref			
Daily-Monthly	918	23.6%	2980	76.4%	0.49	(0.46-0.54)			2492	83.6%	488	16.4%	0.81	(0.72-0.92)		

© 2021 Richards JE et al. *JAMA Health Forum.*

**Depressive Symptoms† (PHQ-9)**																
None-minimal	10864	16.3%	55870	83.7%	Ref				43535	77.9%	12335	22.1%	Ref			
Mild	6326	15.5%	34515	84.5%	1.06	(1.02-1.10)			27998	81.1%	6517	18.9%	0.82	(0.79-0.85)		
Moderate	4153	15.6%	22520	84.4%	1.05	(1.01-1.10)			18610	82.6%	3910	17.4%	0.74	(0.71-0.78)		
Moderate-Severe	2423	15.3%	13376	84.7%	1.07	(1.02-1.13)			11230	84.0%	2146	16.0%	0.67	(0.63-0.72)		
Severe	1428	15.4%	7842	84.6%	1.07	(1.00-1.14)			6767	86.3%	1075	13.7%	0.56	(0.51-0.62)		
**Suicidal ideation† (PHQ-9 Q9)**																
Not at all	21687	15.8%	115639	84.2%	Ref				92280	79.8%	23359	20.2%	Ref			
Several days	2288	15.6%	12423	84.4%	1.02	(0.97-1.07)			10673	85.9%	1750	14.1%	0.65	(0.61-0.69)		
More than half	642	15.7%	3458	84.3%	1.01	(0.92-1.10)			2970	85.9%	488	14.1%	0.65	(0.58-0.73)		
Nearly every day	357	16.8%	1771	83.2%	0.93	(0.82-1.05)			1549	87.5%	222	12.5%	0.57	(0.48-0.67)		
**Anxiety Symptoms† (GAD-2)**																
Negative	16640	16.0%	87433	84.0%	Ref				68876	78.8%	18557	21.2%	Ref			
Positive	8409	15.2%	46937	84.8%	1.06	(1.03-1.10)			39496	84.1%	7441	15.9%	0.70	(0.67-0.73)		
**Depression Diagnosis††**																
No	12766	17.0%	62120	83.0%	Ref		Ref		47972	77.2%	14148	22.8%	Ref		Ref	
Yes	12909	15.0%	73061	85.0%	1.16	(1.13-1.20)	1.06	(1.02-1.10)	61053	83.6%	12008	16.4%	0.67	(0.64-0.70)	0.87	(0.83-0.92)
**Anxiety Diagnosis††**																
No	13377	17.3%	63881	82.7%	Ref		Ref		49351	77.3%	14530	22.7%	Ref		Ref	
Yes	12298	14.7%	71300	85.3%	1.21	(1.18-1.25)	1.14	(1.10-1.18)	59674	83.7%	11626	16.3%	0.66	(0.63-0.69)	0.86	(0.82-0.91)
**Serious Mental Illness Diagnosis††**																
No	24837	15.9%	130955	84.1%	Ref		Ref		105250	80.4%	25705	19.6%	Ref		Ref	
Yes	838	16.5%	4226	83.5%	0.96	(0.88-1.05)	0.94	(0.86-1.04)	3775	89.3%	451	10.7%	0.49	(0.42-0.57)	0.53	(0.45-0.62)
**Substance Use Disorder Diagnosis††**																
No	22496	15.6%	121469	84.4%	Ref		Ref		97768	80.5%	23701	19.5%	Ref		Ref	
Yes	3179	18.8%	13712	81.2%	0.80	(0.76-0.84)	0.77	(0.73-0.81)	11257	82.1%	2455	17.9%	0.90	(0.83-0.97)	0.94	(0.86-1.03)
**Suicide Attempt Diagnosis††**																
No	25579	16.0%	134778	84.0%	Ref		Ref		108648	80.6%	26130	19.4%	Ref		Ref	
Yes	96	19.2%	403	80.8%	0.80	(0.61-1.04)	0.78	(0.59-1.04)	377	93.5%	26	6.5%	0.29	(0.14-0.61)	0.41	(0.19-0.87)
**Charlson Score‡**																
0-1	17970	15.5%	97975	84.5%	Ref		Ref		80006	81.7%	17969	18.3%	Ref		Ref	
2+	4226	16.5%	21320	83.5%	0.93	(0.89-0.97)	1.01	(0.97-1.06)	17145	80.4%	4175	19.6%	1.08	(1.02-1.15)	0.97	(0.91-1.04)

*Not enrolled insurance category includes patients of a Spokane-based healthcare system acquired by KPWA during the study period.
**"Other" race and ethnicity was used to broadly capture all other write-in responses to questions about that do not fall into one of the broader categories, such as "Irish," "Ashkenazi Jewish," or "human race."
***Based on a condensed version of the 2013 National Center for Health Statistics (NCHS) county urban-rural categorization. Missing for 466 visits.
†Recorded via self-report on the MH monitoring questionnaire; see eTable 1 for a summary of non-response.
††Diagnosis in year prior; serious mental illness diagnoses include bipolar, schizophrenia, other psychosis or personality disorders.
‡Missing for 19365 visits without ambulatory or inpatient encounters in which to observe comorbidities during 365 days prior to this visit.
‡‡Adjusted for age, sex, race/ethnicity, rural/urban, diagnoses in year prior††, Charlson score category‡ and prior year enrollment category (not enrolled, <365 days, 356+days).

© 2021 Richards JE et al. *JAMA Health Forum.*

eTable 3. Response and reported access to a standardized question about firearm access in the outpatient mental health specialty setting, observed rates and likelihood of response across demographic and clinical characteristics at the visit-level (using all visits in the study period)

	Responded to the firearm question?								Reported firearm access?							
	Observed Rates N=327,632				Unadjusted N=327,632		Adjusted‡‡ N=313,548		Observed N=290,765				Unadjusted N=290,765		Adjusted‡‡ N=278,080	
	No (11.3%)		Yes (88.7%)		OR	(95% CI)	OR	(95% CI)	No (86.6%)		Yes (13.4%)		OR	95% CI	OR	95% CI
**Age Category**																
18-39	2185	8.9%	22255	91.1%	Ref		Ref		120262	89.5%	14149	10.5%	Ref		Ref	
40-64	1751	9.4%	16815	90.6%	0.93	(0.88-0.99)	0.95	(0.89-1.01)	96705	83.6%	18973	16.4%	1.67	(1.54-1.81)	1.50	(1.39-1.64)
65+	892	11.7%	6733	88.3%	0.82	(0.76-0.90)	0.86	(0.79-0.94)	34800	85.6%	5876	14.4%	1.44	(1.28-1.61)	1.24	(1.09-1.40)
**Sex**																
Female	24354	11.2%	193542	88.8%	Ref		Ref		169905	87.8%	23637	12.2%	Ref		Ref	
Male	12513	11.4%	97209	88.6%	0.98	(0.92-1.04)	1.01	(0.95-1.07)	81848	84.2%	15361	15.8%	1.35	(1.25-1.46)	1.45	(1.34-1.58)
**Race and Ethnicity**																
American Indian/Alaska Native	951	12.3%	6781	87.7%	0.91	(0.73-1.12)	0.91	(0.74-1.13)	5679	83.7%	1102	16.3%	1.17	(0.90-1.51)	1.23	(0.86-1.46)
Asian	1774	11.2%	14009	88.8%	1.00	(0.89-1.13)	0.96	(0.85-1.08)	12868	91.9%	1141	8.1%	0.53	(0.42-0.68)	0.71	(0.56-0.91)
Black	1375	10.1%	12195	89.9%	1.13	(0.99-1.28)	1.06	(0.93-1.22)	11207	91.9%	988	8.1%	0.53	(0.43-0.66)	0.65	(0.51-0.81)
Hawaiian/Pacific Islander	249	8.4%	2720	91.6%	1.39	(1.11-1.73)	1.33	(1.06-1.67)	2371	87.2%	349	12.8%	0.89	(0.61-1.28)	1.07	(0.74-1.56)
Hispanic/Latinx	1788	11.3%	13975	88.7%	0.99	(0.89-1.11)	0.97	(0.87-1.09)	12486	89.3%	1489	10.7%	0.72	(0.60-0.86)	0.83	(0.68-1.01)
other/unk**	1830	11.6%	13910	88.4%	0.97	(0.86-1.08)	0.97	(0.86-1.09)	12353	88.8%	1557	11.2%	0.76	(0.63-0.91)	0.80	(0.66-0.97)
White	28900	11.3%	227175	88.7%	Ref		Ref		194803	85.8%	32372	14.2%	Ref		Ref	
**Insurance***																
Commercial	23330	10.8%	192480	89.2%	Ref				165001	85.7%	27479	14.3%	Ref			
Medicare	9224	12.7%	63460	87.3%	0.83	(0.78-0.90)			55015	86.7%	8445	13.3%	0.92	(0.84-1.02)		
Medicaid	2991	10.8%	24625	89.2%	1.00	(0.91-1.09)			22223	90.2%	2402	9.8%	0.65	(0.56-0.76)		
Not Enrolled	1322	11.5%	10200	88.5%	0.94	(0.84-1.04)			9528	93.4%	672	6.6%	0.42	(0.36-0.50)		
**Rural/Urban*** **																
Urban	15540	11.8%	116082	88.2%	Ref		Ref		107503	92.6%	8579	7.4%	Ref		Ref	
Large Suburban	6168	7.8%	72851	92.2%	1.58	(1.46-1.71)	1.59	(1.47-1.73)	62409	85.7%	10442	14.3%	2.10	(1.90-2.31)	2.13	(1.92-2.36)
Smaller Suburban	13194	12.7%	91039	87.3%	0.92	(0.87-0.98)	0.93	(0.87-0.99)	73936	81.2%	17103	18.8%	2.90	(2.63-3.20)	2.89	(2.61-3.20)
Mostly Rural	1816	16.2%	9412	83.8%	0.69	(0.61-0.79)	0.72	(0.64-0.82)	6724	71.4%	2688	28.6%	5.01	(4.21-5.96)	4.91	(4.09-5.90)
**Alcohol Consumption† (AUDIT-C)**																
Never	10286	8.4%	112020	91.6%	Ref				99870	89.2%	12150	10.8%	Ref			
Low-Level	9431	7.7%	112570	92.3%	1.10	(1.02-1.17)			95343	84.7%	17227	15.3%	1.49	(1.37-1.61)		
Moderate-Level	4744	7.7%	57116	92.3%	1.11	(1.03-1.19)			48705	85.3%	8411	14.7%	1.42	(1.29-1.56)		
High-Level	434	10.1%	3845	89.9%	0.81	(0.70-0.95)			3217	83.7%	628	16.3%	1.60	(1.32-1.95)		
**Cannabis Use†**																
None	15674	7.3%	199250	92.7%	Ref				172455	86.6%	26795	13.4%	Ref			
Weekly-Monthly	4776	7.2%	61979	92.8%	1.02	(0.96-1.09)			54055	87.2%	7924	12.8%	0.94	(0.88-1.02)		
Daily/Almost Daily	3999	13.2%	26376	86.8%	0.52	(0.48-0.56)			22407	85.0%	3969	15.0%	1.14	(1.02-1.27)		
**Other Drug Use†**																
None	22282	7.5%	275844	92.5%	Ref				238583	86.5%	37261	13.5%	Ref			

© 2021 Richards JE et al. *JAMA Health Forum.*

Daily-Monthly	1363	11.9%	10059	88.1%	0.60	(0.55-0.65)			8889	88.4%	1170	11.6%	0.84	(0.74-0.96)		
**Depressive Symptoms† (PHQ-9)**																
None-minimal	8486	11.4%	66091	88.6%	Ref				57530	87.0%	8561	13.0%	Ref			
Mild	9756	11.0%	78932	89.0%	1.04	(0.99-1.09)			68207	86.4%	10725	13.6%	1.06	(1.00-1.12)		
Moderate	8232	11.1%	66189	88.9%	1.03	(0.97-1.09)			56931	86.0%	9258	14.0%	1.09	(1.02-1.18)		
Moderate-Severe	5746	11.1%	46201	88.9%	1.03	(0.97-1.10)			39868	86.3%	6333	13.7%	1.07	(0.98-1.16)		
Severe	4009	10.9%	32822	89.1%	1.05	(0.98-1.13)			28755	87.6%	4067	12.4%	0.95	(0.85-1.06)		
**Suicidal ideation† (PHQ-9 Q9)**																
Not at all	26456	11.0%	213978	89.0%	Ref				183709	85.9%	30269	14.1%	Ref			
Several days	6250	11.4%	48528	88.6%	0.96	(0.91-1.01)			42686	88.0%	5842	12.0%	0.83	(0.77-0.90)		
More than half	2084	11.5%	16038	88.5%	0.95	(0.88-1.03)			14312	89.2%	1726	10.8%	0.73	(0.66-0.81)		
Nearly every day	1449	11.4%	11223	88.6%	0.96	0.87-1.05)			10206	90.9%	1017	9.1%	0.60	(0.51-0.71)		
**Anxiety Symptoms† (GAD-2)**																
Negative	17007	11.5%	131212	88.5%	Ref				113508	86.5%	17704	13.5%	Ref			
Positive	19630	11.0%	158646	89.0%	1.05	(1.00-1.09)			137478	86.7%	21168	13.3%	0.99	(0.94-1.04)		
**Depression Diagnosis††**																
No	17007	11.5%	131212	88.5%	Ref		Ref		72978	86.5%	11407	13.5%	Ref		Ref	
Yes	19630	11.0%	158646	89.0%	1.11	(1.05-1.18)	1.02	(0.96-1.08)	178789	86.6%	27591	13.4%	0.99	(0.92-1.06)	0.84	(0.78-0.91)
**Anxiety Diagnosis††**																
No	5912	12.8%	40133	87.2%	Ref		Ref		34692	86.4%	5441	13.6%	Ref		Ref	
Yes	30955	11.0%	250632	89.0%	1.19	(1.12-1.27)	1.16	(1.09-1.24)	217075	86.6%	33557	13.4%	0.99	(0.91-1.07)	0.97	(0.89-1.06)
**Serious Mental Illness Diagnosis††**																
No	26813	10.7%	224540	89.3%	Ref		Ref		191791	85.4%	32749	14.6%	Ref		Ref	
Yes	10054	13.2%	66225	86.8%	0.79	(0.73-0.84)	0.81	(0.75-0.87)	59976	90.6%	6249	9.4%	0.61	(0.54-0.69)	0.53	(0.47-0.60)
**Substance Use Disorder Diagnosis††**																
No	29274	11.0%	237486	89.0%	Ref		Ref		205050	86.3%	32436	13.7%	Ref		Ref	
Yes	7593	12.5%	53279	87.5%	0.86	(0.82-0.91)	0.86	(0.81-0.92)	46717	87.7%	6562	12.3%	0.89	(0.81-0.97)	0.94	(0.85-1.03)
**Suicide Attempt Diagnosis††**																
No	36208	11.3%	284754	88.7%	Ref		Ref		246046	86.4%	38708	13.6%	Ref		Ref	
Yes	659	9.9%	6011	90.1%	1.16	(0.98-1.37)	1.23	(1.03-1.46)	5721	95.2%	290	4.8%	0.32	(0.23-0.46)	0.39	(0.27-0.55)
**Charlson Score‡**																
0-1	30525	11.2%	242736	88.8%	Ref		Ref		210679	86.8%	32057	13.2%	Ref		Ref	
2+	5090	12.2%	36706	87.8 %	0.91	(0.83-0.99)	0.97	(0.89-1.06)	30886	84.1%	5820	15.9%	1.24	(1.11-1.38)	1.05	(0.94-1.18)

*Sex missing for 5 visits; Not enrolled insurance category includes patients of a Spokane-based healthcare system acquired by KPWA during the study period.
**"Other" race and ethnicity was used to broadly capture all other write-in responses to questions about that do not fall into one of the broader categories, such as "Irish," "Ashkenazi Jewish," or "human race."
***Based on a condensed version of the 2013 National Center for Health Statistics (NCHS) county urban-rural categorization. Missing for 1521 visits.
†Recorded via self-report on the MH monitoring questionnaire; see eTable 1 for a summary of non-response.
††Diagnosis in year prior; serious mental illness diagnoses include bipolar, schizophrenia, other psychosis or personality disorders.
‡Missing for 12575 visits without ambulatory or inpatient encounters in which to observe comorbidities during 365 days prior to this visit.
‡‡Adjusted for age, sex, race/ethnicity, rural/urban, diagnoses in year prior††, Charlson score category‡ and prior year enrollment category (not enrolled, <365 days, 356+days).

© 2021 Richards JE et al. *JAMA Health Forum.*

eTable 4. Consistency of patient-reported firearm access among patients with two or more visits during the study period, stratified by the number of visits and interval between the first and last visit during the study period

	*Response to the firearm question?				†Reported firearm access?			
	**N**	Never	Sometimes	Always	**N**	Never	Sometimes	Always
**Number of Visits**								
Two	17916	3.7%	20.3%	76.0%	16544	78.6%	8.8%	12.6%
Three	9935	1.5%	27.2%	71.3%	9251	77.0%	12.2%	10.8%
Four	6663	0.8%	31.8%	67.3%	6107	76.2%	14.0%	9.8%
Five	4716	0.8%	34.8%	64.4%	4321	77.0%	14.7%	8.3%
Six-Ten	11408	0.7%	42.1%	57.2%	10288	75.9%	17.6%	6.5%
Eleven-Twenty	6287	0.5%	57.0%	42.5%	5488	75.0%	21.5%	3.5%
Over Twenty	3589	0.1%	75.3%	24.6%	2916	72.9%	25.1%	2.0%
**Interval between first and last visit**								
1-7 days (within 1 week)	871	3.2%	17.9%	78.9%	791	82.2%	4.7%	13.1%
8-30 days (1 week-1 month)	5009	2.5%	18.8%	78.7%	4759	80.5%	6.1%	13.3%
31-90 days (1-3 months)	9219	2.1%	21.7%	76.2%	8725	78.8%	9.0%	12.2%
91-180 days (3-6 months)	7354	2.2%	29.3%	68.5%	6648	78.4%	11.6%	10.0%
181-365 days (6 months-1 year)	9830	1.8%	33.2%	65.0%	8928	77.5%	14.0%	8.5%
366-720 days (1-2 years)	12650	1.4%	39.2%	59.4%	11234	75.3%	16.5%	8.2%
720 days to 1460 days (2-4 years)	15581	1.0%	49.5%	49.4%	13830	73.8%	20.4%	5.8%

*Among patients in our sample who received the mental health monitoring questionnaire at least twice (N=60,514).
†Among patients in our sample who received the mental health monitoring tool and answered the question about firearm access at least twice (N=54,915).

© 2021 Richards JE et al. *JAMA Health Forum.*
